# Combined effects of theta-burst stimulation with transcranial direct current stimulation of the prefrontal cortex: study protocol of a randomized, double-blinded, sham-controlled trial using ^99m^Tc-ECD SPECT

**DOI:** 10.47626/2237-6089-2020-0110

**Published:** 2021-12-10

**Authors:** Laís B. Razza, Carlos A. Buchpiguel, Stefanie De Smet, Izio Klein, Chris Baeken, Ricardo Galhardoni, Marie-Anne Vanderhasselt, André R. Brunoni

**Affiliations:** 1 Hospital das Clínicas Faculdade de Medicina Universidade de São Paulo São Paulo SP Brazil Serviço Interdisciplinar de Neuromodulação, Laboratório de Neurociências (LIM-27), Departamento e Instituto de Psiquiatria, Hospital das Clínicas, Faculdade de Medicina, Universidade de São Paulo (USP), São Paulo, SP, Brazil.; 2 Departamento de Medicina Interna Faculdade de Medicina USP São Paulo SP Brazil Departamento de Medicina Interna, Faculdade de Medicina, USP, São Paulo, SP, Brazil.; 3 Department of Head and Skin, Psychiatry and Medical Psychology Ghent University Hospital Ghent University Ghent Belgium Department of Head and Skin, Psychiatry and Medical Psychology, Ghent University Hospital, Ghent University, Ghent, Belgium.; 4 Ghent Experimental Psychiatry (GHEP) Lab Ghent Belgium Ghent Experimental Psychiatry (GHEP) Lab, Ghent, Belgium.; 5 Department of Experimental Clinical and Health Psychology Ghent University Ghent Belgium Department of Experimental Clinical and Health Psychology, Ghent University, Ghent, Belgium.; 6 Escola de Medicina Universidade Cidade de São Paulo São Paulo SP Brazil Escola de Medicina, Universidade Cidade de São Paulo (UNICID), São Paulo, SP, Brazil.; 7 Departamento de Neurologia USP São Paulo SP Brazil Centro de Dor, LIM-62, Departamento de Neurologia, USP, São Paulo, SP, Brazil.; 8 Instituto Nacional de Biomarcadores em Neuropsiquiatria Faculdade de Medicina USP São Paulo SP Brazil Instituto Nacional de Biomarcadores em Neuropsiquiatria (INBioN), LIM-27, Departamento e Instituto de Psiquiatria, Faculdade de Medicina, USP, São Paulo, SP, Brazil.; 9 Hospital Universitário USP São Paulo SP Brazil Hospital Universitário, USP, São Paulo, SP, Brazil.

**Keywords:** Combined effects, non-invasive brain stimulation, prefrontal cortex, spect, cognition, salivary cortisol

## Abstract

**Introduction:**

Non-invasive brain stimulation (NIBS) as monotherapy has been increasingly used to enhance the activity of brain networks. However, it is unclear whether a combination of distinct NIBS approaches could enhance prefrontal cortical (PFC) activity.

**Objective:**

We propose to investigate the combined and standalone effects of two NIBS modalities on the PFC through a working memory task, single photon emission computed tomography (SPECT), and salivary cortisol. We hypothesize that the combined protocol will provoke greater changes in the collected measures compared to the remining protocols.

**Methods:**

A randomized, double-blind, sham-controlled, full-factorial design will be conducted. The effects of transcranial direct current stimulation (tDCS) and intermittent theta-burst stimulation (iTBS) will be investigated over four different sessions (sham tDCS + sham iTBS, anodal tDCS + sham iTBS, anodal tDCS + active iTBS and sham tDCS + active iTBS) in 30 healthy adult volunteers. A 99mTc-ethylene cysteine dimer (99mTC-ECD) will be administered during the NIBS session and neuroimaging will be acquired within one hour. Salivary cortisol will be collected before and after each session and an n-back working memory task will be applied after the end of each NIBS session. The outcomes will be cerebral perfusion alterations (99mTC-ECD SPECT), accuracy and reaction time in the n-back task, and changes in salivary cortisol level.

**Conclusion:**

The results from this trial can guide future therapeutic protocols for NIBS treatments stimulating the PFC by demonstrating that the combination of NIBS techniques is feasible, tolerable, and can lead to greater enhancement of PFC activity.

## Introduction

Non-invasive brain stimulation (NIBS) interventions are non-pharmacological and non-psychotherapeutic techniques that use electric currents to treat psychiatric and neurological disorders.^1 , 2^ The main forms of NIBS are repetitive transcranial magnetic stimulation (rTMS) and transcranial direct current stimulation (tDCS). rTMS is based on magnetically-induced fields that are generated from a coil placed over one’s head, causing a discharge of potent electric currents that induce action potentials (APs).^3^ A relatively novel rTMS protocol is called theta-burst stimulation (TBS), which consists of a series of pulses, usually between 3 to 5, at 50 Hz (called a ‘‘burst’’), delivered at a frequency of 5 Hz. This frequency coincides with the theta frequency band of electroencephalography.^2 , 4^ In turn, tDCS uses a low-intensity current delivered to the brain through two electrodes placed on the head. Although tDCS does not generate APs *per se,* its current is capable of modulating cortical excitability towards depolarization (anodal tDCS) or hyperpolarization (cathodal tDCS).^5^

The effects of these NIBS interventions over the dorsolateral prefrontal cortex (DLPFC) have been individually investigated as treatments for several psychiatric conditions and for cognitive enhancement,^6 - 8^ presenting promising results mainly for major depressive disorder (MDD).^9 - 11^ However, the standalone effects of tDCS on cognition and MDD are only moderate.^12 - 14^ Likewise, the moderate effects and cost-benefit profile of rTMS are issues that limit its widespread use as a mainstream treatment modality, leading the field to develop novel personalized approaches.^15^

Based on the rationale that tDCS modulates spontaneous activity via subthreshold alterations of resting membrane potentials, several studies have investigated whether tDCS could potentiate the neural system and thereby enhance the effects of other interventions.^16 , 17^ TDCS applied over the DLPFC combined with other therapies such as antidepressant drugs^18^ and working memory training^19^ have already been evaluated for depression, showing promising results for the combined protocols. Notwithstanding, the effects of tDCS concomitantly applied with different patterns of rTMS (inhibitory and excitatory protocols) have already been investigated over the primary motor cortex (M1) of healthy volunteers.^20 , 21^ These results showed that combined protocols presented greater effects on the amplitude of motor-evoked potentials (towards inhibition and excitation) in comparison to each NIBS technique alone. Besides, to the best of our knowledge only one trial has already evaluated the effects of concomitant application of tDCS and intermittent TBS (iTBS) over the PFC. This study investigated the effects of combined tDCS-iTBS on stress, showing no evidence that combined tDCS-iTBS, compared to iTBS alone, attenuates the psychophysiological stress response in healthy subjects.^22^

Therefore, considering the modest effects of these NIBS techniques as monotherapy and the advances in investigation of combining NIBS therapies, the main aim of this study is to assess the standalone and combined effects of a sub and a suprathreshold NIBS protocol associated with neural facilitation - anodal tDCS and iTBS over the left DLPFC. We propose a phase-I study, with multimodal assessments including 99mTc-ethylene cysteine dimer (ECD) brain SPECT (single photon emission computed tomography), working memory tasks, and salivary cortisol. These three measures were chosen to evaluate direct and indirect PFC functioning, respectively. Regarding the indirect measures, the cognitive task and cortisol levels are associated, respectively, with the PFC cortical-subcortical network and the hypothalamic-pituitary-adrenal (HPA) system, which presents its sensitivity significantly influenced by rTMS effects over the PFC.^23^ Therefore, we hypothesize that the combined protocol will be associated with greater changes in cerebral perfusion, as well as cognitive performance and salivary cortisol levels, than the other protocols (NIBS alone and sham).

## Material and methods

### Design

We propose a phase-I, randomized, double-blind, within-subjects, full-factorial study design, which is efficient for testing the standalone and combined effects of tDCS and iTBS.

### Participants

We will recruit 30 volunteers of both genders, aged from 18 to 45 years, right-handed, and with no prior or present mental or neurological disorders and/or clinical diseases. A pre-screening email will be administered beforehand.

Meeting the pre-screening criteria, each participant will be screened in person for past and/or current psychiatric or neurological diagnoses by trained psychologists with the aid of structured interview questionnaires based on the DSM-5 diagnostic criteria. The evaluators will apply the Portuguese version of the Mini International Neuropsychiatric Interview,^24^ the Hamilton Depression Rating Scale (HAMD),^25^ and the Beck Depression Inventory (BDI),^26^ aiming to investigate, respectively, the presence of any cognitive dysfunction or mood disorders, such as anxiety or depression. The Positive Affect Negative Affect Scale (PANAS)^27^ will also be applied as previous studies suggest that the scale might be a proxy for clinical improvement of depression,^28^ a condition in which the DLPFC is the main target for treatment using NIBS. The self-assessment tool for depression (BDI) will be administered first and further hetero-evaluation (HAMD) will be conducted in order to confirm the emotional state of the participants. Finally, the Edinburgh handedness inventory^29^ will be administered to confirm laterality.

Exclusion criteria are: 1) any contraindication for the techniques to be administered (i.e., tDCS, rTMS, SPECT, and magnetic resonance imaging [MRI]), such as metal implants in the cranial region, including metal plates in the skull and implants in the central nervous system; 2) habitual smokers (more than 10 cigarettes per day), or abuse of/dependence on other drugs; 3) pregnant or planned pregnancy during the study; 4) use of psychoactive drugs, including antidepressant drugs, benzodiazepines and Z-drugs; 5) serious neurological or clinical conditions; 6) conditions that prevent weekly attendance at the service; 7) HAMD score > 7; 8) BDI score > 10.

### Procedure

At the beginning of the study, participants will be screened for participation and asked to give written informed consent. The study is in accordance with the Declaration of Helsinki and was approved by the Ethics Committee at the São Paulo University Hospital das Clínicas (HCFMUSP) and registered on the Plataforma Brasil database (CAAE: 89310918.8.0000.0068).

Participants will be randomized in accordance with a computer-generated list at www.randomizer.org and allocation will be conducted by an assistant not involved in the project. Participants will be asked to come to the research center five times. In line with previous studies of (non)combined NIBS protocols, there will be a one-week interval between the sessions to ensure elimination of carryover effects.^20^ On the first day, all participants will undergo a 3 Tesla MRI (GE, 750W system integrated within a 3.0 Tesla General Electric PET/MRI scanner) scan during which we will acquire T1-weighted and T2-weighted sequences (repetition time [RT] = 1900 ms, echo time [ET] = 2.2 ms, flip angle = 9°, 176 slices/volume, slice thickness = 0.8 mm). MRI acquisition is performed beforehand to target the right and left DLPFC via a neuronavigation system (Brainsight, Rogue Resolutions, Inc). Both left and right DLPFC will be located in each participant by reverse coregistration from the MNI152 stereotaxic coordinates (x - 38, y + 44, z + 26 and x + 38, y + 44, z + 26, respectively) and marked on a cap. This method has been identified as optimal on the basis of clinical outcomes and whole brain functional connectivity.^30 , 31^ After neuronavigation, all other sessions will take place in a well-controlled laboratory environment at the Institute of Psychiatry - HCFMUSP, São Paulo, Brazil. The same procedures will be applied in each of the four sessions, except for neuronavigation, which will only be conducted during the first session.

**Figure 1:**
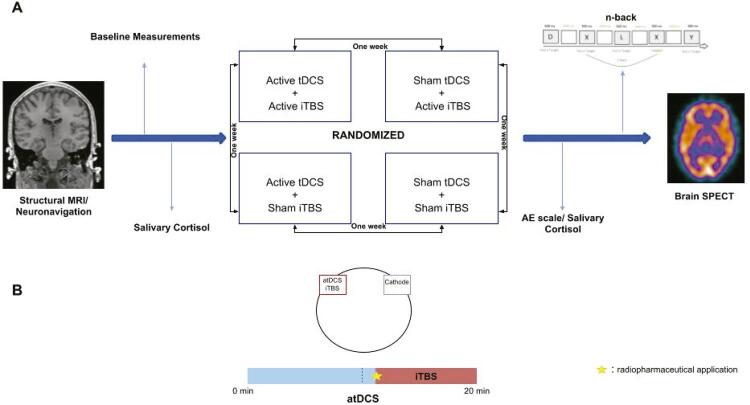
Protocol that will be applied. A) Sequence of the data acquisition and group allocation. B) Noninvasive brain stimulation protocol. atDCS = anodal transcranial direct current stimulation; AE = adverse effects; iTBS = intermittent Theta-burst stimulation; min = minutes; MRI = magnetic resonance imaging; SPECT = single photon emission computed tomography.

Before the start of each of the four experimental sessions, participants will be asked to answer a few baseline measurements such as the HAMD, BDI, PANAS, and Hamilton Anxiety Inventory (HAI) and a visual analogue scale (VAS). These will essentially be applied to identify participants’ brain state before the NIBS session and can also serve as potential moderators of the NIBS effects. Afterward, the volunteers’ resting motor threshold^32^ will be determined using a MagVenture device (MagPro X100, Denmark) and a figure of eight coil. Salivary cortisol will be collected after the motor threshold measurement and before venous access is obtained for administration of the 99mTc-ECD radiotracer. After venous access, subjects will undergo a neurostimulation session ( Figure 1A ). First, the tDCS device will be switched on. The tDCS session will last 20 minutes. However, the tDCS protocol will be applied alone for the first eleven minutes and then, for the last 9 minutes of the neurostimulation session, the iTBS protocol over the left DLPFC will be applied concomitantly. The radiotracer (99mTC-ECD) will be injected right after the start of the iTBS protocol ( Figure 1B ).

VAS and salivary cortisol will be collected immediately after the end of the session. For the VAS scale, participants will be asked to manually indicate on horizontal 10 cm lines whether they are experiencing any current pain. ‘Zero’ means ‘no pain’ and ‘ten’ means ‘pain as bad as it could possibly be’. To ensure the safety of both NIBS techniques alone and their combination, an Adverse Effects Scale^33^ will be assessed. This scale consists of identifying the produced effects and measuring them on a scale from 1 to 4 points, being 1 – absent, 2 – mild, 3 – moderate and 4 – severe. The working memory task (n-back) will be applied after measurement of adverse effects.

All of the procedures described above will take place at the Institute of Psychiatry - HCFMUSP. However, after the end of the working memory task, the volunteers will be taken to the Nuclear Medicine Center - HCFMUSP, where the SPECT images will be acquired by a trained team.

### SPECT

Volunteers will be asked to fast 6 hours prior to the SPECT exam. Initially, an injection of the 99mTc-ECD radiotracer with activity of 20 mCi (555 MBq) will be administered during the NIBS session. Images will be acquired up to 60 minutes after administration in SPECT equipment (630 model GE, Milwaukee, WI, USA) consisting of a dual-head gamma camera with dedicated collimator for brain studies (Fan beam). SPECT images will be acquired sequentially with a standardized protocol using a 2.5x zoom factor, 128 x 128 matrix, and acquisition of 240 3/3° projections (two 360° turns) every 20 seconds. The photopic will be centered at 140keV, with a window at 10%. Processing will be performed using an interactive reconstruction method (OSEM) with Butterworth filter, with a cutoff frequency of 0.57, and serial number of 10. The number of interactions used will be 20, with 40 subsets. In addition, we will use the T1 and T2 sequences to correct the data for partial volume effects.

### NIBS protocols

Based on previous studies with healthy volunteers^34^ and depressed patients^13 , 14^ using tDCS, the anode will be placed on the left and the cathode over the right DLPFC cortices (bilateral montage), according to the neuronavigation coordinates. A current of 2mA will be applied using 25cm^2^ saline-soaked sponges. All sessions will last 20 minutes and will be performed using a Neuroconn DC-Stimulator MR device (Neuroconn GmBH, Ilmenau, Alemanha) that has a “study mode” and allows for automatic double-blinding according to an imputed code. The sham protocol will consist of a brief active period of 30 seconds fade-in and 30 seconds fade-out at the beginning and end of the session. The randomized codes for all four sessions for both tDCS and iTBS interventions will be stored in sealed, opaque envelopes. Only one person not directly associated with the project will have access to them. For each session, this person will write the codes on a note and deliver it to the technician responsible for the administrations.

ITBS will be performed over the left DLPFC, with a stimulation target located based on neuronavigation. The TMS coil will be placed above the anodal tDCS electrode (coil-scalp-distance: approximately 5mm) and positioned 45 degrees relative to the midline. Stimulation will consist of 54 cycles of 10 triplet bursts with train duration of 2s and an interval of 8s between two successive trains (total of 1620 pulses; Huang et al., 2005^4^ ), at 110% of resting motor threshold. The resting motor threshold will be operationalized as the minimum TMS intensity necessary to visually yield a motor evoked potential in the right abductor pollicis brevis muscle in 5 out of 10 successive attempts.^35^ Based on these parameters, the iTBS protocol will last approximately 9 minutes. All sham and active stimulations will be performed using the MagVenture device. We will employ the Magventure coil (Cool – B65 Active/Placebo), that also has a ‘study mode’, allowing sham or active TMS sessions depending on the numerical imputed code. Thus, from the imputed randomized code, the device will display on the screen which side of the coil should be used. The coil has two identical, symmetrical sides and the sham protocol is additively identical to the active but does not produce electromagnetic pulses.

### Working memory (n-back) task

The n-back will be programmed in E-prime 2.0 software (Psychology Software, Tools Inc Pittsburgh, Pennsylvania, USA). Images will be presented on a 15-inch LCD computer screen and participants will be seated at a distance of approximately 50 cm from the screen. The visual stimulus consists of letters of the alphabet (A to Z) that will appear individually and randomly on the computer screen. Two conditions will be applied, 0-back and 2-back. The tasks will present 3 blocks of 30 letters, each one being displayed on the screen for 500ms, with an interstimulus interval of 3000 ms. A correct response occurs when the subject identifies letter ‘X’ (0-back task) and the same stimuli presented two positions before (2-back task). Response time will also be measured. Higher accuracy values will represent improvement, whereas lower (including negative) response times will represent faster response. This is the same protocol previously used by our group.^8 , 36^

Finally, the n-back task will not be applied before the NIBS session to avoid learning effects. Moreover, n-back performance after the sham protocol will be used as the reference value in our statistical comparison and the randomized and counter-balanced NIBS protocols will allow us to statistically evaluate learning effects by introducing order as a variable in our statistical models. This methodology was applied based on previous studies evaluating working memory performance with NIBS intervention.^37^

### Salivary cortisol

Saliva samples will be collected using salivettes with an insert containing a sterile polyester swab, yielding a clear and particle-free sample. They will be used according to the manufacturer’s instructions (Sarstedt G & Co, Numbrecht, Germany). The salivettes will be centrifuged at 500 rpm for 2 hours, and the filtrates will be stored frozen (−20C) until analysis, which will be performed with standard ELISA kits from Enzo Life Sciences Inc, New York, USA. The samples will be thawed and individually recentrifuged before the analysis.

### Statistical analyses

Sample size was calculated using G*Power 3.1 software.^38^ Since this is an original study and no a-priori power analysis is provided, we estimate a moderate *f* – size effect (0.25). The analysis considered four groups, 4 measurements, correlation between repeated measures of 0.5, alpha of 0.05 and power of 0.85, resulting in a sample size of 28 participants. Considering possible losses, we estimate recruitment of 30 subjects (120 observations).

The Shapiro-Wilk test will be applied to identify the sample distribution. Assuming a normal sample, we will perform analysis of variance (ANOVA) having ‘group’ as independent variable and ‘area of activation’ (observed in the left DLPFC) as dependent variable. The Kruskal-Wallis test will be performed in case of a non-parametric sample. The SPECT images will be available in orthogonal cuts in the transverse, sagittal, and coronal planes (DICOM format). Images will be analyzed using the Statistical Parametric Mapping software (SPM12; Wellcome Department of Imaging Neuroscience, London, UK), executed in Matlab Version 2020 (MathWorks, Natick, MA). We will use SPM to form a statistical map according to the statistical threshold to identify differences among the groups. In-house scripts will be used for partial volume correction procedures based on structural MRI. The SPM images will be subtracted from each group to verify areas of functional activation.

ANOVA or its non-parametric extension will be applied for n-back and salivary cortisol, with ‘group’ as independent variable for both outcomes and ‘cortisol level’ (for salivary cortisol) and accuracy/response time (for working memory performance) as ‘dependent variables’. The associations between n-back task performance (accuracy and response time)/salivary cortisol level and cerebral activation will be estimated with linear regression. We will perform pathway analyses to verify whether cerebral activation is directly associated with the results of working memory performance and cortisol salivary level or is measured by these findings. We will also statistically investigate the possible learning effects of the repetitive application of the working memory task by grouping participants’ scores according to the chronological order of NIBS intervention assignments.

The mood scales administered before each NIBS session (such as HAMD, BDI, PANAS, and HAI) will be used to investigate major mood oscillation between sessions. If significant mood changes (p < 0.05) are found, we will use these measures as potential moderators of the main outcomes of this study. Moreover, the VAS will be used to investigate any significant change in pain intensity before and after each session as well as between NIBS sessions. Finally, ANOVA will also be applied to investigate adverse effects (dependent variable) across groups (independent variable).

## Discussion

This study will be a phase-I trial designed to assess the combined and standalone effects of the tDCS and iTBS interventions over the PFC using multimodal assessments, comprising neuroimaging, cognitive tests, and salivary cortisol measures, which will be essential to evaluate the effects of concomitant NIBS administration, as well as to enhance knowledge regarding the effects of individual application of the techniques.

Considering the previous studies investigating the effects of two NIBS interventions^20 , 21 , 39^ and neuroimaging studies evaluating the online effects of tDCS over the DLPFC (17 subjects for two groups, with a total of 34 observations),^40^ our study presents the largest sample recruited to date. Besides, the within-subject full-factorial design will allow us to analyze a total of 120 observations. This sample decreases the chances of type I and type II errors, compared to previous studies.

Regarding the combined protocol adopted in our study, although application of dual facilitatory protocol stimulation can increase the chance of provoking homeostatic plasticity effects,^21^ this approach was chosen since previous studies have shown that induction of both LTP (long-term potentiation) or LTD (long-term depression) by burst stimulation depends on the membrane potential of the postsynaptic neurons and that the effects of stimulation can be modified by external hyper/depolarization.^20 , 41^ Besides, according to the literature, tDCS effects are improved by a concept known as ‘functional targeting,’^17^ in which neuronal networks that are already activated might be more stimulated compared to non-primed ones. Following these findings, the rationale behind the concurrent application of tDCS and iTBS is that DC current will probably bias iTBS effects.^20^ Moreover, it is important to underscore that the protocol for this study is slightly different from the ‘priming’ protocols of the previous studies with TMS,^42^ since here the stimulation protocols will be applied concomitantly and both NIBS protocols are associated with neuronal facilitation.

Along these lines, 99mTc-ECD SPECT was the imaging methodology of choice since it can measure direct and online brain perfusion of the NIBS protocols and the 99mTc-ECD radiopharmaceutical has low intrasubject variability.^43^ Thus, we decided to perform SPECT instead of other neuroimaging methods because 1) SPECT is a well-known method; 2) compared with the Positron Emission Tomography technique, SPECT uses longer-lived, more easily obtained radioisotopes, allowing us to apply the cognition task before acquisition of neuroimaging^44^ ; 3) combining two NIBS techniques and MRI simultaneously is extremely challenging; and 4) SPECT provides a direct measure of cerebral perfusion, in contrast to the fMRI technique, which is based on the BOLD signal and has low test-retest reliability at rest for tDCS studies.^45^ In fact, brain perfusion SPECT has already been applied to investigate the acute effects on regional cerebral blood flow (rCBF) of low-frequency (LF) and HF-rTMS in depressed patients,^46^ as well as to evaluate the effects of rTMS and antidepressant drugs in patients with Parkinson disease and depression^47^ pre/post treatments. Both studies showed directional, significant regional cerebral blood flow (rCBF) alterations after NIBS intervention over the PFC.

Moreover, the other assessments (working memory performance and salivary cortisol) will expand the findings of this study. The PFC and its neural network are associated with several cognitive functions^6^ and play an important role in emotional responses, processing and regulating stress.^48^ For instance, the DLPFC shows activation when sadness is suppressed voluntarily^49^ and co-activation with the amygdala during emotion reappraisal.^50^ In stressful events, the amygdala may activate other brain regions and, respectively, increase cortisol levels. Studies have already shown that a single session of tDCS over the DLPFC can change cortisol levels during negative visual stimuli.^48 , 51^ Analyzing cortisol measures in our study will allow us to identify if the iTBS and tDCS protocols (as monotherapy or combined) play an important role in changes to cortisol levels compared to sham protocol, as well as to compare these measures to brain activity in the different protocols. The same will be conducted using the working memory performance measure. In fact, instead of other cognitive domains such as attention, executive functions, or social cognition that are also related to the PFC function, working memory was chosen as the cognitive outcome of this study because this domain is the most investigated in NIBS trials to date^52^ and because previous trials have also shown that working memory is a potential marker for depression.^36^ Moreover, acquisition of SPECT neuroimaging within one hour of radiotracer injection makes it difficult to investigate any other measures.

Finally, the effects of the combination of tDCS and rTMS protocols have already been investigated for patients with stroke and tinnitus, for instance. In general, the results of the combined protocol showed significant clinical improvement when compared to the interventions as monotherapy.^53 , 54^ Accordingly, demonstrating the combined effects of two NIBS interventions over the PFC is important for further directions for therapeutic protocols given that both tDCS and iTBS techniques are limited as monotherapies for the treatment of PFC dysfunctions. For instance, standard rTMS protocols show a response rate of only 35% for depressed patients.^55^ Although the iTBS protocol is a recent U.S. Food and Drug Administration (FDA) approved technique for depression,^30^ meta-analyses indicate that its response rate is similar to standard rTMS protocols.^10 , 11^ Likewise, tDCS trials are still reporting heterogeneous results.^14 , 56 , 57^

### Limitations

First, we will not compare the effect of iTBS and cathodal tDCS that have also been shown to increase cortical excitability.^21^ Second, some technical limitations regarding the SPECT exam should be underscored: 1) SPECT measures the CBF, which does not generally exactly reflect brain metabolism, being a better surrogate for brain activity compared with cerebral perfusion; 2) although SPECT is a consolidated neuroimaging method, it presents poorer contrast and spatial resolution when compared to Positron Emission Tomography (PET), for instance; and 3) the within-subject design may introduce learning effects in working memory tasks.

## Conclusion

This phase-I study is a randomized, sham-controlled, double-blind, full-factorial, within-subjects design trial, which will investigate the combined effects of tDCS and iTBS protocols over the PFC of healthy volunteers using 99mTc-ECD SPECT, cognitive tests, and cortisol measures. The results from this trial have the potential to optimize the future therapeutics protocols for NIBS treatments targeting the PFC for disorders such as major depression.
